# How Trust Makes a Difference: The Impact of the First Wave of the COVID-19 Pandemic on Life Satisfaction in Germany

**DOI:** 10.1007/s11482-021-09956-0

**Published:** 2021-08-02

**Authors:** Felix Bittmann

**Affiliations:** Leibniz Institute for Educational Trajectories, Wilhelmsplatz 3, 96047 Bamberg, Germany

**Keywords:** COVID-19 lockdown, Germany, Institutional trust, Life satisfaction, I14, I31

## Abstract

The extraordinary COVID-19 pandemic is one of the most severe disruptions of human life since the end of World War II, even in rich and industrialized countries like Germany. The introduction of a rather comprehensive “lockdown” and the restriction of multiple basic civil rights have affected the population in many areas of life, like employment, economic prosperity, health and trust in public institutions. The question arises how life satisfaction is influenced by these measures in detail and whether there are interactions between institutional trust, life satisfaction and time of crisis. Fixed-effect regression analyses using German National Educational Panel Study (NEPS) data demonstrate that life satisfaction has fallen sharply after the onset of the crisis and that interaction effects with institutional trust are present. Individuals with low levels of pre-crisis trust in institutions like the government, courts or the media report a stronger decrease of satisfaction than individuals with higher levels of trust. We believe that these results are relevant to explain the role of institutions in times of crisis and might serve as foundations for interventions to strengthen trust and increase overall satisfaction.

## Introduction

The COVID-19 pandemic can be described as one of the biggest global crises since World War II, as it affects basically every human being, regardless of their place of residence, social status or political affiliation (Lohse, 2020). It has caused considerable dislocation even in rich western industrialized nations like Germany. Even if Germany, by acting swiftly and prudently, managed to overcome the first wave of the crisis compared to similar European countries with below-average infection and mortality rates, the entire society and social life was massively affected (Wieler et al., 2020). Whereas in the first phase following the initial infection on January 27, 2020, the number of infected persons in the federal state of Bavaria rose only slowly, the spread accelerated considerably thereafter, so that a large number of restrictions were imposed from March 16 onwards (Möhring et al., 2020). It seems likely that these measures have had a significant impact on the social and psychological well-being of the population and effects on overall life satisfaction are to be expected (Diener, 2012). The "lockdown" has considerably restricted social contacts and reduced the meeting of friends and relatives to a minimum. Increased work from home also reduced contact with colleagues, and residents of nursing homes were downright isolated, as contact from outsiders was prevented. It can be concluded that the first wave of the COVID-19 pandemic in Germany is one of the biggest crises in post-war history and can be classified as a period effect that affects all members of a population (Kosloski, 1986; Zacher & Rudolph, 2020). Clearly, this might affect satisfaction of the population but there is more to that. Since it is obvious that many aspects of the crisis are either mediated, explained or experienced through social institutions like the political system, the executive branch or the media, the second question arises how the perception and the trust into these institutions interacts with the crisis situation (Sibley et al., 2020). One can plausibly argue that institutions have gained power in the pandemic as they had to enact and enforce virtually unprecedented measures, like a comprehensive and nationwide “lockdown”. Is it conceivable that people who have higher trust in these institutions are able to deal better with the negative effects of the crisis since they believe that the challenges are accepted by responsible, capable and benevolent forces? We believe that these questions deserve more attention. To summarize, the present contribution attempts to answer two major research question: first, how did the overall satisfaction change after the onset of the COVID-19 pandemic and the “lockdown”? Second, do individuals react differently to the crisis, depending on their pre-crisis trust in public institutions?

## Assumptions and Hypotheses

In general, social institutions can be described as “integrated systems of rules that structure social interactions" (Hodgson, 2006, p. 501) that exist in any human society. Humans as social beings who live together in smaller or larger communities require rules, norms and some forms of laws to facilitate order and function properly. Through historical and ethnographic studies, researchers have identified numerous social institutions that take these responsibilities (Macionis & Gerber, 2011). While this short overview cannot give a complete picture and a proper introduction to the definition and function of an institution, it is central to understand that they are necessary to structure and regulate human behavior and are above the individual since they are the sum of the behavior of many human beings (emergence) and can involve all areas of life (Greif, 2006). To give a short overview of widespread contemporary institutions: the government, the legal system, churches (religion), marriage or education. All modern human societies have some forms of institutions as they become more important the larger and interconnected these societies become due to growing complexity and interdependencies.

The second major construct for the present research question is *satisfaction*, also often termed *well-being* or simply *happiness*. The study of satisfaction is a fast-growing area in the social sciences, economics and health-studies since it relates to many areas of life. It affects every human being and is central to personal development (Frey & Stutzer, 2005). Not only have some constitutions described the “pursuit of happiness” an unalienable human right (Cahn, 1952) while some others have moved on to actually measure the condition of a country in terms of happiness, instead of economic terms, like with the GDP (Karma Ura, 2012). While it is rather obvious to see why the study of happiness is relevant, it is much more difficult to actually find a commonly agreed on definition as not only the measurement but also the conceptualization of the term is challenging (Cummins, 1995; Medvedev & Landhuis, 2018). In addition to that, it is already well known that happiness is affected by numerous factors: family, health, education, economic resources, employment, psychological traits and many more (Cummins, 2000, 2012). While it is straightforward to predict severe crises (like the global 2008 recession) influence satisfaction negatively (Habibov & Afandi, 2015), it is interesting to see that the long-term effects often cannot be explained by short-term negative effects (as unemployment) and affect different social groups differently in the long run (Clench-Aas & Holte, 2017). Giving a more complete overview is not feasible at this place, however, it is crucial to explain that institutions and especially the trust an individual has in these social institutions affects happiness.

Since most areas of human life are somehow regulated by institutions (for example, the legal system and the police, the educational system, press and media), we can argue that individuals with trust in these institutions have also higher values of satisfaction (Mueller, 2009). As institutions are often very powerful, difficult to ignore and deviations from the norms punishable, believing that these institutions are benevolent and helpful is relevant for the personal well-being. On the other hand, individuals who deeply distrust these institutions are probably not very satisfied since they usually do not have the option to abandon and ignore them since they are present in most areas of life. Especially interesting is the interaction between the onset of crisis through the COVID-19 pandemic and institutions. We have good reasons to believe that the role of some institutions has grown since then. After COVID-19 appeared, governments were required to take action, enact new laws and regulations and protect the population from the emerging danger. Hence this process also affected the legal system, the police and the health system since these are the executing institutions, enforcing laws and supporting people when they fall sick and need assistance. But many more are involved, for example the media, which have the obligation to report independently about the crisis, monitor the actions of the government and other institutions (hence the role as “fourth estate”) and put all events into perspective. Individuals who have trust in these institutions probably believe that the crisis can be overcome since many agencies are involved to protect the country and do their best to find a solution.

Based on these theoretical considerations we propose three testable hypotheses. First, we assume that in general and even before the onset of the COVID-19 crisis in Germany, individuals with higher institutional trust did have higher satisfaction, on average (hypothesis one). Second, satisfaction has decreased after the onset of the COVID-19 crisis and the nationwide “lockdown” (hypothesis two). Third, there is an interaction effect present between crisis and trust; the satisfaction of individuals reporting high values of trust before the crisis has declined less sharply than of individuals with lower values of trust (hypothesis three).

These assumptions are supported by previous research findings. First, the correlation between institutional trust and satisfaction has been empirically demonstrated in various cultural settings (Ciziceno & Travaglino, 2019; Hudson, 2006; Macchia & Plagnol, 2019; Mueller, 2009; Zhang & Zhang, 2015). Regarding the second hypothesis, there are recent studies available that demonstrate, for multiple countries, that satisfaction is negatively affected. For Germany, we observe negative effects (Brandt et al., 2021; Möhring et al., 2020; Zacher & Rudolph, 2020) and especially women and parents with small children suffer from the consequences of the “lockdown” (Huebener et al., 2020). Similar outcomes are found for the UK (Foa et al., 2020; Shen & Bartram, 2020), Spain (Blasco-Belled et al., 2020), Italy (Maugeri et al., 2020) China (Zhang et al., 2020) or other countries (Gawrych et al., 2021; Özmen et al., 2021). With respect to the third question, fewer findings are available. Only one published study from China considers institutional trust as a mediator between crisis and satisfaction and reports a positive relationship (Sun & Lu, 2020). However, it should be made transparent that the authors consider trust as a subdimension of *social capital* and the comparability to the present question is probably not ideal. Hence we believe that the following analyses have the potential to unearth new and innovative results that might be of great relevance. If it can be demonstrated that institutional trust and negative influence of the crisis are related, this might offer the potential for structural interventions to strengthen trust in relevant institutions and uphold high levels of satisfaction in the population.

Summarized, the present contribution adds to the recent and fast growing literature about the social and psychological consequences of the COVID-19 pandemic in various ways. By using panel data, effects of the pandemic can be judged with a clear and defined temporal order, which is helpful to put recent developments into perspective.

## Data, Methods and Operationalization

### Data and Sample

The foundation of all further analyses is the German National Educational Panel Study (NEPS), starting cohort 6 (Blossfeld et al., 2011).^1^ This long-running panel study with a focus on the role of education throughout the life course serves as a proper data source for various reasons. First, the panel, which started in 2008 and continues with annual surveys of adult participants, provides a long history of variables that allow temporal tracking. This makes it possible to recognize general time trends within the data. Second, the dataset not only includes information about education but a wide range of psychometric and sociodemographic information like working history and career development, marriages and divorces, information about life satisfaction and other relevant psychological items, health and various other aspects which were identified as related to life satisfaction. Starting cohort six includes adult participants from all stages in the life course and thus provides data about a rather representative sample of the Federal Republic of Germany. Selective attrition, dropout and nonresponse can be ameliorated using imputation techniques, which are discussed in more detail below.

For the analyses especially three waves of the survey are of interest: waves ten to 12. Wave 12 is the COVID-19 extra survey which was not part of the regular survey plan but added spontaneously to gather information about the situation within the COVID-19 crisis at the height of the lockdown in Germany.^2^ Therefore, this survey is much smaller than the regular waves and only contains a base set of variables and some additional items tailored for the crisis situation. This survey was conducted online (CASI) from May 13, 2020 to June 22, 2020 (Weiß, 2020). This means the items were gathered directly after the case number started to fall again. Regular participants from wave eleven were first contacted by mail (sent mid-May 2020) and invited to take part in an online survey. They also received an email reminder three weeks afterwards (if an email contact was available in the database and if they did not complete the survey yet). However, this survey generated a much lower response rate than usual waves (realization rate 27.3%) which can be attributed to the online and self-administered form of the study (no interviewer present), the unexpected date (unplanned extra-survey), the special situation (crisis situation) and the much lower response time (less than six weeks to participate in the study). This clearly is a drawback for analyses. Some remedies are described in more detail below.

The surveys in wave ten and eleven were administered via telephone or with an interviewer (CATI or CAPI) from August 2017 to March 2018 (wave ten) and from September 2018 to April 2019 (wave eleven). The realization rates of the surveys are 87.6% (wave ten) and 88.2% (wave eleven) (Malina et al., 2018; Steinwede & Aust, 2019). In absolute numbers, these are 8,125, 7,695, and 2,771 subjects who participated in waves ten to 12. For the following analyses participants are removed that have missing values on satisfaction or institutional trust in wave ten since this critical information should not be imputed for precise estimates.

### Operationalization

**Fig. 1:**
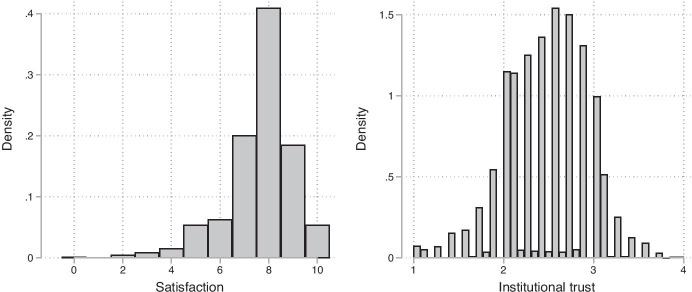
Histograms of satisfaction and institutional trust. Source: NEPS SC6, N = 8119, not imputed data

The central dependent variable, overall life satisfaction, is operationalized using the following item: “First of all, I would like to ask you some questions about your current satisfaction with various aspects of your life. Please answer on a scale from 0 to 10. '0' means that you are ‚completely unsatisfied’, '10' means that you are ‚completely satisfied’. You can graduate your answer with the numbers [integers] in between. All in all, how satisfied are you with your life at the moment?”. This variable with eleven levels is posed in all waves of the survey and can thus be traced over time. For the analysis of the given research question and overall life satisfaction, the NEPS recommends using this single variable and not generating a compound score. The main independent variable is institutional trust, which is measured in wave ten using seven items, which report the overall trust of a person in institutions (federal government, parliament, federal constitutional court, the European Union, banks, the press and television. Each item has four categories from one (no trust at all) to four (very much trust); an index score is generated by computing the arithmetic mean with a fine reliability (Cronbach’s Alpha = 0.802) and a rather normally distributed shape (see Fig. 1). We believe that this item is adequate since all institutions included in the items are to some extent related to the crisis situation, may it be political, social, economical or medial. Additional statistics to assess the quality of this construct are provided in the appendix (Table 3). Note that this instrument is an adaption of two other instruments previously used by ALLBUS and SOEP (Beierlein et al., 2012; Fehr et al., 2003), which are two other high-quality and long-running German population surveys.

### Strategy of Analysis

As the COVID-19 pandemic is a global and “random” external shock with unclear causes, investigating the causal effects of this event is, statistically speaking, rather straightforward. As there are no confounders for this “treatment” it is not necessary to include classical control variables in the following analyses. The inclusion of additional variables might even cause bias since they might “explain away” the overall effects of the pandemic (overcontrol bias) (Elwert & Winship, 2014), which is not advised since the total effect is to be estimated. Hence, it must be emphasized that no mediation analyses are of interest and adding more variables might underestimate the total effect (Bartram, 2019). However, we are interested in estimating the interaction with institutional trust, that is considered a (rather) time-constant trait. We thus opt for fixed-effect (FE) panel regressions with two time points (eleven and 12). Such a model computes the *within*-person change, which is beneficial since it removes the influence of time-constant influence automatically (for example, the effects of gender, year of birth or migration background). The regression equation is as follows:

1 $${Satisfaction}_{it}=\beta_0+\,{Wave}_{it}{\beta }_{1}+{Trust}_{i}{\beta }_{2}+Wave_{it}\,\times\,{Trust}_{i}{\beta }_{3}+{\epsilon }_{it}$$

*Wave* is a binary indicator (1 for the COVID-19 survey, 0 for the previous survey wave), *Trust* is an ordinal variable with four levels (reporting quartiles) and the third term is the interaction of both variables. Notice that the term *Trust* is dropped automatically from the FE-model since it is time constant, however, this is without relevance since the interaction term captures the effect of interest (the group-specific change of satisfaction). Note that this statistical model is equivalent to a first-difference (FD) estimator since there are only two waves included (Brüderl & Ludwig, 2015).

Even though the main analytical model is rather undemanding, panel attrition and non-random dropout are major challenges. Hence, we propose an imputation design to account for potential selective dropout. There are good reasons to assume that some social groups have a higher chance of not participating in the COVID-19 extra survey: older participants might be reluctant to take part in the survey online without the assistance of an interviewer; people with small children at home might have less time since they have to monitor their children constantly as schools and other childcare institutions are closed. If these groups also have different satisfaction values, which is a plausible assumption, bias emerges. Multiple imputation with chained equations (MICE) is a widespread statistical option to account for these factors (Allison, 2002). The idea is to generate an imputation model that includes potential predictors of participation. By doing so, bias is attenuated (Azur et al., 2011). Since there are strong predictors available (like previous satisfaction), imputing the dependent variable is acceptable (Sullivan et al., 2015). We select the following variables for the imputation model: satisfaction (wave ten, eleven and 12), gender, age, institutional trust (wave ten), health, logarithmized (total) monthly household income (after tax), self-rated health (TNS Infratest Sozialforschung et al., 2012), whether German is the native language of the respondent, the number of children younger than 18 years living in the household, whether the mother and father of the participant were born in Germany, education with four levels (low education *Hauptschulabschluss* / intermediate education *Mittlere Reife*, higher education eligibility *(Fach-)Abitur* / any tertiary education), marital status (single / married / divorced / widowed), employment status (retired or not employed / regular employee / civil servant / self-employed / other employment) and the region of residence (South / East / West). Unless specified in detail, the information for these variables is taken from the most recent time point available prior to wave 12. Anchor is wave ten and only respondents who participated in this wave are included in the subsequent analyses. By doing so we avoid imputing information of people that dropped out a longer time before as their information might not be reliable. We generate 100 complete datasets after a burn-in sequence of 60 rounds. Statistical output of the imputation procedure (convergence, creation of values within a plausible range) was inspected and approved. All analyses are computed in Stata 16.1, complete do-files are available on request.

## Empirical Analyses

First, we give a summary of the most central descriptive statistics to demonstrate the social and demographic composition of the sample. This is relevant to judge how similar the NEPS sample is to the overall German population, which can be achieved by a comparison with official statistics (Table 1).

**Table 1:** Descriptive summary of the analytical sample

	Mean	SD	Min	Max	Imputed
Satisfaction W10	7.64	1.4	0	10	0^*^
Satisfaction W11	7.63	1.4	0	10	0.093
Satisfaction W12 (COVID-19)	6.63	1.9	0	10	0.678
Institutional trust W10	2.47	0.5	1	4	0^*^
Female respondent	0.51	0.5	0	1	0
Age in 2020	56.68	10.7	34	76	0
Self-rated health	3.71	0.8	1	5	< 0.05
Log. total household income	8.09	0.5	6	10	< 0.05
German not mother tongue	0.06	0.2	0	1	< 0.05
Number of children in the household	0.42	0.8	0	17	0.093
Mother not born in Germany	0.10	0.3	0	1	< 0.05
Father not born in Germany	0.11	0.3	0	1	< 0.05
Level of education					
Low education	0.19	0.4	0	1	< 0.05
Intermediate education	0.32	0.5	0	1	< 0.05
Higher education eligibility	0.19	0.4	0	1	< 0.05
Tertiary education	0.30	0.5	0	1	< 0.05
Marital status					
Single	0.16	0.4	0	1	< 0.05
Married	0.71	0.5	0	1	< 0.05
Divorced	0.09	0.3	0	1	< 0.05
Widowed	0.04	0.2	0	1	< 0.05
Employment status					
Not employed / Retired	0.25	0.4	0	1	< 0.05
Regular employee	0.56	0.5	0	1	< 0.05
Civil servant	0.06	0.2	0	1	< 0.05
Self-employed	0.10	0.3	0	1	< 0.05
Other employment	0.03	0.2	0	1	< 0.05
Region of residence					< 0.05
Southern Germany	0.28	0.4	0	1	0.112
Eastern Germany	0.21	0.4	0	1	0.112
Western Germany	0.52	0.5	0	1	0.112

Source: NEPS SC6, imputed data (M = 100). Variables marked with an asterisks are not imputed by design as all cases with missing information were removed before

While the gender distribution is almost equal, the average age in the sample is clearly higher than in the overall population (44.5 years) (Statistisches Bundesamt, 2019). This means that the sample is not representative of the overall population, which is probably due to the longer running nature of the NEPS and the selective panel attrition. It must also be taken into account that the panel will naturally mature over time since no refreshment samples were drawn for many years and the youngest participants in the sample are 34 years old. Therefore, the following analyses cannot give information of the effects for the youngest cohort in Germany. The sample is also more highly educated than the population. In the sample, about 49% of all respondents have at least some form of higher education eligibility, while in the German population, this value is only about 33% in 2019. In addition to the numerical values we provide a graphical depiction of the distribution of satisfaction and institutional trust, measured in wave ten (Fig. 1).

Regarding the share of imputed values, the only critical value is satisfaction in the COVID-19 survey. Here, about 68% of all information is imputed. Not imputed at all are the values for satisfaction in wave ten and trust in wave ten due to the sample selection process.

**Fig. 2:**
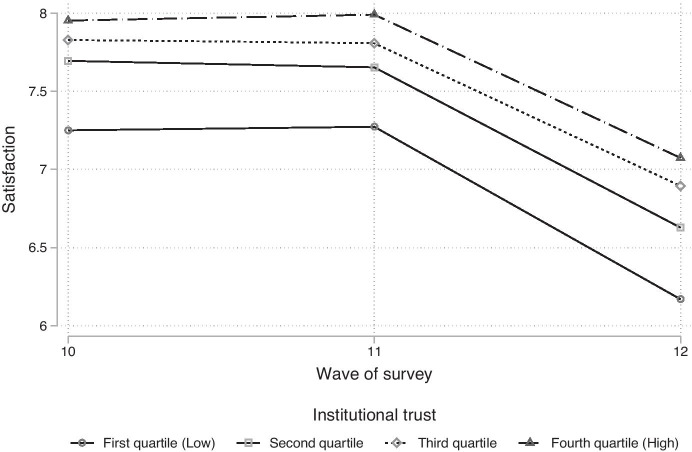
Temporal development of life satisfaction by level of trust. Source: NEPS SC6, imputed data (M = 100)

Next, we provide a rather simple descriptive figure to see how life satisfaction changes over the three waves of interest. Here, only means are computed, separately for each of the four quartiles of institutional trust (Fig. 2). The results clearly indicate that life satisfaction is rather stable before the onset of the pandemic since there are almost no changes between wave ten and eleven. Furthermore, we observe that apparently trust and satisfaction are correlated since groups with higher trust do have higher values of satisfaction, on average. This trend is stable over time. To furthermore test this statistically, a simple linear regression model is computed for wave eleven with satisfaction as the dependent variable and trust quartiles as the sole predictor (not depicted). Since the p-value of the overall model fit (omnibus test) is very small (N = 8,119, p < 0.001, imputed data), this proves statistically that institutional trust and life satisfaction are correlated and people with higher trust do have higher values for satisfaction, on average (before the onset of the crisis). This finding is in line with the descriptive results (Fig. 2) and theoretical expectations, consequently, hypothesis one is accepted.

We continue with the main analytical models, which are fixed-effect panel regressions. We present three models: the first includes the wave dummy as the sole explanatory variable to test how satisfaction changes, on average, between wave eleven and 12. This effect is the total effect, associated with the COVID-19 changes. The second model adds the interaction term between the wave dummy and the four quartile groups of trust. These effects quantify whether the four different groups are affected differently by the COVID-19 effect. The third model utilizes the original and continuous measurement of trust as a robustness check. Results are presented in Table 2.

**Table 2:** FE-regression results for satisfaction

	M1	M2	M3
Wave 11	Ref	Ref	Ref
Wave 12	-1.007^***^ (0.0370)	-1.105^***^ (0.0735)	-1.453^***^ (0.219)
Wave 12 × 1. Quartile Trust		Ref	
Wave 12 × 2. Quartile Trust		0.0783 (0.0771)	
Wave 12 × 3. Quartile Trust		0.191^*^ (0.0943)	
Wave 12 × 4. Quartile Trust		0.187^#^ (0.112)	
Wave 12 × Trust (continuous)			0.181^*^ (0.0842)
Constant	7.633^***^ (0.0152)	7.633^***^ (0.0152)	7.633^***^ (0.0152)
Observations	16,238	16,238	16,238
Individuals	8119	8119	8119

Source: NEPS SC6, imputed data (M = 100). Standard errors in parentheses. ^#^ *p* < 0.10, ^*^ *p* < 0.05, ^**^ *p* < 0.01, ^***^ *p* < 0.001

In model M1, the constant is 7.63, which is the overall life satisfaction in wave eleven (also see Table 1, which gives the same number). The wave dummy effect is -1.01 and statistically highly significant. It means that life satisfaction has dropped by about one point in the COVID-19 survey in comparison to the previous wave, which signifies a large change. This clearly underlines that life satisfaction has decreased, on average, in the COVID-19 survey, which is in line with theoretical expectations. Hence, we accept hypothesis two. When we continue with model M2, we add the interaction terms of the quartiles of trust. The first quartile (lowest institutional trust) is the category of reference. We notice that all point estimates are positive, hence, the other groups have smaller changes in satisfaction, on average. The p-values indicate that not all effects are statistically significant on the 5% level, yet the trends are clear. The point estimates indicate that the higher the trust, the less satisfaction decreases in the COVID-19 survey. The concrete numerical interpretation is as follows: an individual with the lowest trust decreases their satisfaction by 1.105 points (this effect is included in the overall wave dummy effect since it is the category of reference). A person with the highest trust has a reduction of about 0.918 points (-1.105 + 0.187), which is a less drastic decline than for an individual with the lowest trust. This finding is in line with theoretical expectations, hence we accept hypothesis three. Apparently, the change of satisfaction partly depends on pre-crisis trust in institutions. As a robustness check, we do not use the coarsened quartiles of trust in M3 but the original continuous variable. While this model is less simple to interpret, the statistical effect is significant on the 5% level and in line with M2. Hence we conclude that generating four levels of trust, which is an arbitrary decision, does not much affect the results.

To further strengthen the validity of the findings, we compute the same table using listwise deletion instead of imputation. The coefficients are comparable and conclusions do not change much. The regression table is included in the appendix (Table 4). Note that from a statistical point of view the imputed results should be less biased and more correct as they account for potential selective dropout.

## Discussion

As the results indicate, people with higher institutional trust have higher satisfaction, on average before the onset of the crisis. These findings are in line with theoretical expectations since human society is grounded on various institutions that govern, regulate and enable the social life of millions of people within a country. When people believe that these institutions are useful and benevolent, this should affect their satisfaction positively since they cannot be avoided as they regulate almost all aspects of life. Note that this finding is a correlation, so it is not possible to state that high trust in institutions actually *causes* high satisfaction. Next, the overall effects of the pandemic are of interest. As the findings clearly indicate, these are negative, as one would expect. The death of thousands of people, the fear of a new and horrific disease and the closing of schools, workplaces, restaurants and many other establishments of daily life has been undoubtedly a drastic and unprecedented event for the German society in the last 50 years. As the pandemic can be regarded as a “random” shock that affects every person, we can probably consider these results as causal effects since there are no classical confounders involved that could generate spurious findings. As the results are based on FE-estimates, they should be especially robust since the within-person change is computed. By now, as already outlined above, many other studies from various cultural settings have confirmed these negative effects of the pandemic. Note that these are total effects averaged over the entire sample so we cannot a.) pinpoint what exactly is the mechanism behind them and b.) whether some sociodemographic groups are affected differently. Follow-up studies might want to research these open questions in more detail.

Finally, the greatest interest lies in the interaction between institutional trust and satisfaction. As the results indicate, these interactions are demonstrable and people with low values of trust suffer more than people with high trust, which cannot be explained by pre-treatment differences between the groups. This finding is also in line with expectations since institutions have gained power and relevance in the time of crisis. The government and parliament are required to react quickly, enact measurements to curb the spread of the disease and plan ahead to protect the population, while also taking many other interests, like economic ones, into account. The media and press are required to report independently and monitor the work of the government and other agencies. Courts need to constantly review the new policies and decide whether they are compatible with the German constitution, which is especially important since basic rights were restricted as well. Apparently, people who can trust these institutions can deal better with the consequences of the pandemic and keep higher hopes. They probably believe that these institutions will be able to protect them and the country from the worst consequences and take care of the emerging problems. However, people who already distrust these institutions might despair more quickly since they do not believe that relevant agencies can deal with the crisis appropriately or might even exacerbate them. From a statistical point of view, these findings are probably rather causal since trust was measured before the onset of the crisis so the arrow of causality points in a clear direction. Of course, it is obvious that the crisis itself might affect trust differently and some people will gain trust, others will lose it, which is, however, a different research question and deserves more attention in following analyses.

Finally, the limitations of the study should be discussed to outline the limits and the scope of the analyses. First, the measurement of the dependent variable *satisfaction* utilizes only a single item, which is usually not the optimal option to capture a high degree of detail and variance and give a complete picture of the overall satisfaction. This is a limitation due to the data available. However, we believe this is not a severe restriction since comparable studies have also used this form of operationalization successfully (Brandt et al., 2021; Möhring et al., 2020).

Second, as discussed before, the rate of participation in wave 12 is much lower than in the previous waves due to some reasons discussed above. Multiple imputation is utilized to address this issue since many time-constant auxiliary variables are available from previous waves. By comparing imputed and not-imputed results we can estimate that the total bias due to the selective dropout is probably not too severe. However, there is a related aspect, that is, the special circumstances of the wave 12 survey. As the response setting is different and the time-frame much shorter, it is unclear whether this introduces systematic (measurement) error which might affect the results.

Third, as outlined with the descriptive statistics above, the analytical sample is not representative of the German population or even the NEPS SC6 sample in the first wave due to the long-running nature of the survey (starting in 2008/09), selective dropout, panel attrition and the absence of refreshment samples. Hence, the sample omits the youngest age groups, has a higher average age and is selective since it contains more highly educated individuals than the German population. Consequently, it is not straightforward to generalize the findings and the external validity is affected. However, as there are no data available from official statistics to test these questions, we cannot quantify the strength of this bias.

Fourth, as always with observational data, the numbers reported do probably not reflect pure causal effects. While there are many reasons to believe the findings are of high quality, the restrictions outlined above must be taken into account.

## Conclusion

Analyses with German NEPS SC6 data demonstrate that overall life satisfaction of the population dropped massively in 2020 in comparison to previous surveys and the main assumption is that this reduction is a consequence of the global COVID-19 pandemic, which affected Europe and Germany drastically. Given the large scale of restrictions of basic civil rights and daily life this result is not surprising and in line with previous findings. Of special interest are detail analyses which investigate how pre-crisis institutional trust and the crisis situation interact. The results show that the lower the trust in institutions, the larger the decline of satisfaction. This finding is according to expectations since people who believe that relevant institutions like the government, courts or the press cannot deal with the crisis adequately might lose hope and confidence more quickly, which in turn affects their satisfaction. We believe that these findings are important to understand how and why public institutions are relevant in difficult times and need to address the concerns of the population. This finding also hints that building trust in these institutions might also affect satisfaction positively and it is of greatest relevance to constantly work on the quality and reputation of these central institutions. We believe that building and strengthening trust in public institutions might be a feasible option to increase overall satisfaction. While our findings are observational and are hence not able to give pure causal findings, we hope that they are a first contribution to an in-depth investigation. We welcome replication studies, especially from different social and cultural contexts to test the overall external validity of the results.

## Appendix

**Table 3:** Statistical fit of institutional trust score

Dependent variable	Fitted	Predicted	Residual	R^2^	MC	MC^2^
Federal government	0.458	0.338	0.120	0.739	0.859	0.739
Parliament	0.468	0.373	0.095	0.797	0.893	0.797
Federal Court	0.585	0.221	0.364	0.378	0.615	0.378
EU	0.479	0.172	0.307	0.359	0.599	0.359
Banks	0.439	0.051	0.388	0.117	0.341	0.117
Press	0.493	0.091	0.402	0.184	0.429	0.184
TV	0.404	0.049	0.355	0.121	0.349	0.121
Overall				**0.894**		
Cronbach’s Alpha	0.802					
McDonald’s Omega	0.795					
RMSEA^*^	0.161	(Root mean squared error of approximation)				
CFI^*^	0.851	(Comparative fit index)				
TLI^*^	0.777	(Tucker-Lewis index)				
SRMR^*^	0.088	(Standardized root mean squared residual)				
CD^*^	0.894	(Coefficient of determination)				

Source: NEPS SC6, not imputed data, wave ten. Statistics marked with an asterisk are computed from a confirmatory factor analysis using a SEM-framework

**Table 4:** FE-regression results (listwise deletion)

	M1	M2	M3
Wave 11	Ref	Ref	Ref
Wave 12	-0.963^***^ (0.0370)	-1.071^***^ (0.0779)	-1.416^***^ (0.213)
Wave 12 × 1. Quartile Trust		Ref	
Wave 12 × 2. Quartile Trust		0.0625 (0.102)	
Wave 12 × 3. Quartile Trust		0.227^*^ (0.107)	
Wave 12 × 4. Quartile Trust		0.153 (0.114)	
Wave 12 × Trust (continuous)			0.178^*^ (0.0823)
Constant	7.818^***^ (0.0262)	7.818^***^ (0.0262)	7.818^***^ (0.0262)
Observations	5120	5120	5120
Individuals	2560	2560	2560

NEPS SC6, listwise deletion utilized. Standard errors in parenthese

^#^ *p* < 0.10, ^*^ *p* < 0.05, ^**^ *p* < 0.01, ^***^ *p* < 0.001

## Footnotes

1 *This paper uses data from the National Educational Panel Study (NEPS): Starting Cohort Adults, *doi:10.5157/NEPS:SC6:11.1.0*. From 2008 to 2013, NEPS data was collected as part of the Framework Program for the Promotion of Empirical Educational Research funded by the German Federal Ministry of Education and Research (BMBF). As of 2014, NEPS is carried out by the Leibniz Institute for Educational Trajectories (LIfBi) at the University of Bamberg in cooperation with a nationwide network.*

2 Officially, the COVID-19 survey is not referred to as “wave 12 “ as not to interfere with the regular upcoming wave 12. However, for the sake of brevity, in the following, the SC6 COVID-19 survey is referred to as “wave 12”.
